# Sex differences in sleep architecture and nocturnal oxygenation across obstructive sleep apnea severity: an observational hospital-based study

**DOI:** 10.1186/s13293-026-00951-4

**Published:** 2026-07-07

**Authors:** Meina Wu, Pei Xue, Jinzhu Yan, Christian Benedict

**Affiliations:** 1 Department of Neurology and Sleep Medical Center, Fujian Provincial Governmental Hospital, Fuzhou, China; 2 Department of Pharmaceutical Biosciences, Uppsala University, Uppsala, Sweden

**Keywords:** Obstructive sleep apnea, Sex differences, Sleep architecture, Nocturnal oxygenation, Polysomnography

## Abstract

**Background:**

Sex differences in obstructive sleep apnea (OSA) may extend beyond the apnea–hypopnea index (AHI), the standard measure of OSA severity, and involve differences in sleep architecture and nocturnal oxygenation. We compared these characteristics between women and men across OSA severity categories in a large Chinese clinical cohort.

**Methods:**

This single-center, hospital-based observational study included adults referred for overnight polysomnography at a sleep center in Fujian, China. OSA severity was classified as mild, moderate, or severe according to AHI. Outcomes included total sleep time (TST), sleep stages, sleep fragmentation, nocturnal oxygenation, respiratory event composition, and the relative distribution of oxygen desaturation events between rapid eye movement (REM) and non-REM sleep. Associations between sex, OSA severity, and these outcomes were examined using adjusted generalized linear models.

**Results:**

Among 1,632 patients (mean age 57 years; 41.4% women), women exhibited longer TST, a lower proportion of stage N1 sleep, a higher proportion of stage N3 sleep, and lower sleep fragmentation than men, irrespective of OSA severity (all *p* < .001). Men exhibited a higher proportion of REM sleep than women only in mild OSA (+ 1.46 percentage points, *p* = .009). Women showed a greater predominance of hypopneas than men in moderate and severe OSA (*p* = .02 and *p* < .001, respectively). Women also exhibited lower nadir oxygen saturation (SpO2) values than men across OSA severity categories (*p* = .001). In addition, women demonstrated a greater predominance of REM-related oxygen desaturation events than men in both moderate and severe OSA (both *p* < .001).

**Conclusions:**

Women and men with similar OSA severity exhibited distinct patterns of sleep architecture, respiratory event composition, and nocturnal oxygenation. These findings suggest that characteristics beyond AHI may provide additional information regarding the physiological expression of OSA, although their clinical relevance remains to be determined.

**Supplementary Information:**

The online version contains supplementary material available at https://doi.org/10.1186/s13293-026-00951-4.

## Plain language summary

Obstructive sleep apnea is a condition in which breathing repeatedly stops or becomes restricted during sleep. It can lead to poor sleep quality, reduced oxygen levels, and long-term health problems. Although obstructive sleep apnea affects both women and men, it may not present in exactly the same way in both sexes. We studied 1,632 adults referred for sleep testing at a hospital in China. Each participant underwent an overnight sleep study that measured breathing disturbances, sleep stages, and blood oxygen levels. We then compared women and men across different levels of sleep apnea severity. Women generally slept slightly longer, spent more time in deep sleep, and experienced fewer sleep disruptions than men. Men, in contrast, spent more time in lighter sleep stages and had more frequent brief awakenings during the night. We also found differences in breathing patterns during sleep. In moderate and severe sleep apnea, women experienced a greater proportion of partial breathing reductions (hypopneas), whereas men experienced relatively more complete breathing pauses (apneas). Women also reached lower minimum oxygen levels during sleep than men. In addition, oxygen desaturation events in women were more strongly concentrated during rapid eye movement (REM) sleep, particularly in moderate and severe sleep apnea. Overall, our findings show that women and men with similar sleep apnea severity can differ in sleep quality, breathing patterns, and oxygen levels during sleep. These results suggest that measures beyond the standard severity index may provide additional information about how obstructive sleep apnea is expressed in women and men. Further studies are needed to determine whether these differences have implications for diagnosis, prognosis, or treatment.

**Supplementary Information:**

The online version contains supplementary material available at https://doi.org/10.1186/s13293-026-00951-4.

## Background

Obstructive sleep apnea (OSA) is a sleep-related breathing disorder in which the upper airway repeatedly becomes partially or completely blocked during sleep, leading to disrupted breathing and reduced blood oxygen levels [1]. If untreated, obstructive sleep apnea can not only impair sleep quality but also increase the risk of, e.g., cardiovascular disease [2] and cognitive impairment [3]. Early recognition and diagnosis are therefore important for reducing long-term health consequences.

OSA has historically been considered more common in men, a view reflected in widely used screening tools such as STOP-Bang, where male sex increases predicted risk [4]. However, this assumption is increasingly being challenged [5, 6]. OSA prevalence increases in women during the perimenopausal transition [7], and women more often present with hypopneas rather than apneas, suggesting less pronounced airway collapse and different oxygen desaturation patterns [8–11]. Clinical presentation also differs by sex: men more commonly report snoring and witnessed apneas, whereas women more often report insomnia, fatigue, and mood-related symptoms [12, 13]. These differences may contribute to underdiagnosis in women, as their symptoms are less specific and less likely to meet common screening and referral criteria for OSA, often presenting as insomnia, fatigue, and mood-related complaints rather than loud snoring or witnessed breathing pauses [5, 6].

However, it remains unclear whether these sex differences vary across OSA severity levels, which describe how frequently breathing is interrupted during sleep and how much these interruptions affect blood oxygen levels. Severity is typically classified using the apnea–hypopnea index (AHI), a measure of the number of breathing pauses or shallow breaths per hour of sleep, and is commonly grouped into mild, moderate, and severe obstructive sleep apnea based on increasing frequency of these events [1]. Therefore, this study aimed to examine whether sleep patterns and blood oxygen levels differ between women and men across different levels of OSA in a large clinical cohort from China, where the condition may occur even at lower body mass index due to differences in body structure [14, 15].

## Methods

### Participant selection

**Fig. 1:**
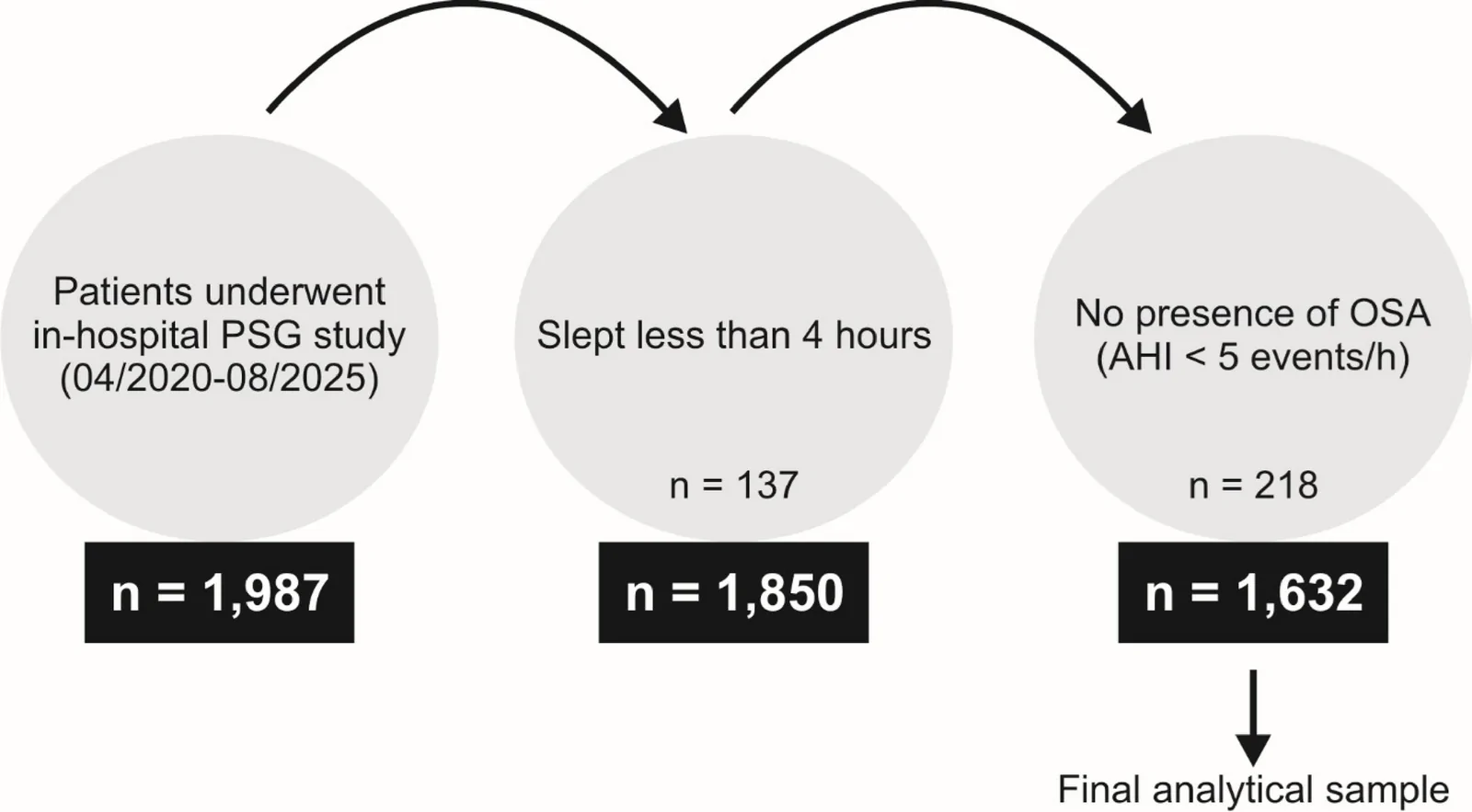
Study flow diagram showing participant selection. Abbreviations: PSG, polysomnography; OSA, obstructive sleep apnea; AHI, apnea–hypopnea index

Between April 2020 and August 2025, 1,987 adult patients (44% women; mean age, 57 years) in Fujian, China, underwent overnight in-hospital polysomnography for OSA evaluation. Participants were predominantly of Han ethnicity (98.3%). As shown in Fig. 1, a total of 137 patients who slept < 4 h were excluded, as such short sleep duration limits reliable assessment of OSA metrics [16]. In addition, 218 patients with AHI < 5 events/h (indicative of absence of OSA, see [17]) were excluded, yielding 1,632 participants. The study was approved by the Institutional Ethics Committee of Fujian Provincial Governmental Hospital, and all participants provided written informed consent.

### Sleep assessment

Participants underwent attended overnight polysomnography in a sleep laboratory using a digital system (Alice 6 LDxS; Philips Respironics, USA). Participants were not permitted to take sleep medication on the night of the study. Standard physiological signals were recorded, including electroencephalography, electrooculography, electromyography, airflow (oronasal thermal sensor and nasal pressure transducer), thoracoabdominal respiratory movements, and pulse oximetry. Sleep stages and respiratory events were scored according to the criteria of the American Academy of Sleep Medicine [16, 17].

Sleep stages, including non-rapid eye movement (non-REM) sleep stages N1, N2, and N3, and rapid eye movement (REM) sleep, were expressed as a percentage of total sleep time (TST). To characterize sex differences in sleep fragmentation, we further calculated the total arousal index, defined as the number of arousals per hour of sleep. An arousal was defined as a sudden shift in electroencephalography frequency lasting at least 3 s, preceded by at least 10 s of stable sleep, and scored according to standard polysomnographic criteria [16, 17].

Respiratory events were defined according to the 2017 scoring rules [16, 17]. OSA was quantified using the AHI, defined as the number of apneas and hypopneas per hour of sleep, and used to determine both presence and severity of the disorder. Hypopneas were scored when airflow was reduced by ≥ 30% for at least 10 s in association with a ≥ 3% oxygen desaturation or an arousal, whereas apneas were defined as a ≥ 90% reduction in airflow lasting at least 10 s. Four respiratory and nocturnal oxygenation outcome variables were derived: the relative proportion of hypopneas (% of apneas and hypopneas), time spent asleep with oxygen saturation < 90% (T < 90% SpO2; minutes), nadir oxygen saturation (Nadir SpO2; %), and the oxygen desaturation index based on ≥ 3% desaturations (ODI-3%; events per hour of sleep).

### Statistical analysis

Analyses were performed using SPSS version 30.0 (IBM Corp., Armonk, NY, USA). Generalized linear models were used to examine whether associations between OSA severity and sleep architecture and nocturnal oxygenation differed by sex. Model distributions and link functions were selected based on outcome distributions (e.g., gamma distribution with log link for skewed variables). For outcomes containing zero values, a constant of 1 was added prior to model fitting to satisfy the distributional requirements of gamma models with a log link. Because this constant was applied uniformly to all observations and was small relative to the observed range of the affected variables, interpretation of the exponentiated parameter estimates as relative differences between groups was unchanged. Interaction terms between sex and OSA severity were included in all models. In the absence of a statistically significant interaction (*p* ≥ .05), models were re-specified to include main effects only. Parameter estimates with standard error [SE] are reported.

Because men and women in the severe OSA group differed in AHI, inverse probability weighting (IPW) based on propensity scores was applied in all primary analyses involving this subgroup. Propensity scores were estimated from a logistic regression model with sex as the grouping variable and AHI as the predictor. The resulting inverse probability weights were used to balance AHI distributions between men and women while retaining all participants. Following weighting, the AHI imbalance in the severe OSA group was substantially reduced (unweighted: women 49.65 ± 1.27 vs. men 55.85 ± 0.88 events/h; standardized mean difference [SMD] = 0.34; weighted: women 54.62 ± 0.78 vs. men 54.07 ± 0.72 events/h; SMD = 0.03). All reported analyses for the severe OSA subgroup were conducted using the weighted sample.

Standardized deviance residuals were examined to identify extreme outliers. For model stabilization, observations with residuals outside the range of − 3 to 3 were excluded. Details of these exclusions, model specifications, and confounder adjustments are provided in Supplementary Table S1.

All tests were two-sided, with *p* < .05 considered statistically significant. This study is reported in accordance with the Strengthening the Reporting of Observational Studies in Epidemiology (STROBE) reporting guideline.

### Confounders

For TST and sleep stage outcomes, models were adjusted for insomnia (yes/no), periodic limb movement index (PLMI), age at investigation, and body mass index (BMI), as these factors may independently influence sleep architecture. Insomnia was defined, based on a structured clinical interview, as difficulty initiating or maintaining sleep or early morning awakening occurring at least three times per week for more than three months, accompanied by daytime symptoms. Participants reporting regular use of prescription hypnotic medication were also classified as having insomnia, regardless of current symptom status, as symptom improvement may reflect ongoing treatment. Information on hypnotic medication use was obtained during the clinical assessment and reflected regular prescribed treatment. In accordance with the sleep laboratory protocol, prescription hypnotic medications were not taken on the PSG night and therefore did not directly influence the recorded sleep study measures. The PLMI was defined as the number of periodic limb movements per hour of sleep, assessed using polysomnography according to standard scoring criteria [17]. BMI was calculated as weight in kilograms divided by height in meters squared. For nocturnal oxygenation outcomes, analyses were additionally adjusted for TST and time spent sleeping in the supine position, as these factors may influence oxygen desaturation severity.

## Results

### Cohort characteristics

Women were generally older than men across all OSA severity categories, whereas BMI tended to be higher in men across all OSA severity categories.Insomnia was more prevalent in women. Respiratory measures reflected expected severity patterns, with AHI, ODI-3%, and T < 90% SpO2 increasing with OSA severity. In the severe OSA category, men exhibited higher AHI values than women, indicating an imbalance in OSA severity between sexes. Because Table 1 presents descriptive statistics from the original unweighted sample, IPW based on AHI was applied in all primary analyses involving severe OSA to balance AHI distributions between women and men while retaining all participants.

### TST (in hours)

A summary of adjusted estimated marginal means (EMMs) for TST and sleep stages by sex and OSA severity is provided in Table 2. For TST, no significant interaction between sex and OSA severity was observed (Wald χ²(2) = 0.19, *p* = .91); therefore, the model was re-specified to include main effects only. In the adjusted analysis, women exhibited longer TST than men, with EMMs that were 0.24 h higher (adjusted EMMs [SE] for men vs. women: 6.47 [0.04] vs. 6.70 [0.04] h; Wald χ²(1) = 17.97, *p* < .001). No significant main effect of OSA severity was observed (adjusted EMMs [SE] for OSA severity: mild, 6.57 [0.06] h; moderate, 6.65 [0.06] h; and severe, 6.55 [0.04] h; Wald χ²(2) = 2.15, *p* = .34).

**Table 1:** Clinical, sleep, and respiratory characteristics by sex and obstructive sleep apnea severity

Characteristic	Mild OSA		Moderate OSA		Severe OSA	
	Women (*n* = 274)	Men (*n* = 256)	Women (*n* = 214)	Men (*n* = 266)	Women (*n* = 187)	Men (*n* = 435)
Age, years	58.0 (12.7)	54.6 (14.8)	62.1 (10.9)	55.4 (15.0)	65.1 (10.7)	54.3 (14.0)
BMI, kg/m^2^	23.1 (3.2)	24.4 (3.0)	24.4 (3.0)	24.9 (3.0)	25.6 (3.6)	27.4 (3.8)
Presence of insomnia, n (%)	210 (76.6)	157 (61.3)	157 (73.4)	140 (52.6)	116 (62.0)	126 (29.0)
PLMI, events/h	16.0 (23.8)	17.9 (26.9)	17.3 (21.6)	18.0 (27.0)	17.9 (23.3)	20.6 (23.3)
Time in bed, h	8.6 (1.2)	8.5 (1.3)	8.7 (1.2)	8.7 (1.2)	8.6 (1.3)	8.6 (1.3)
TST, h	6.8 (1.3)	6.6 (1.3)	6.7 (1.2)	6.6 (1.3)	6.5 (1.2)	6.5 (1.3)
N1, % TST	9.7 (5.2)	12.3 (6.0)	11.6 (6.6)	15.0 (7.4)	15.4 (10.4)	23.3 (13.5)
N2, % TST	57.6 (13.4)	56.2 (11.5)	54.9 (12.0)	54.1 (11.1)	53.4 (12.5)	49.3 (11.3)
N3, % TST	15.7 (11.9)	12.6 (10.4)	16.0 (11.4)	12.4 (10.4)	14.8 (11.2)	10.8 (9.9)
REM, % TST	17.0 (6.5)	18.9 (6.9)	17.5 (7.1)	18.5 (6.7)	16.4 (6.7)	16.5 (6.2)
Supine sleep time, h	4.0 (2.3)	3.4 (2.2)	3.9 (2.2)	3.6 (2.1)	4.0 (2.3)	3.7 (2.3)
Arousals, events/night	50.2 (29.7)	52.9 (30.6)	66.5 (36.0)	74.9 (43.9)	102.1 (80.5)	147 (111)
Total arousal index, events/h	7.4 (4.1)	8.2 (4.7)	10.1 (5.7)	11.4 (6.6)	15.9 (12.2)	22.7 (15.6)
Hypopneas, events/night	51 (19)	50 (23)	105 (41)	97 (40)	171 (87)	120 (87)
Apneas, events/night	13 (14)	14 (13)	39 (34)	49 (38)	152 (131)	241 (165)
Hypopneas, % of apneas and hypopneas	80.1 (18.1)	77.5 (20.1)	73.2 (20.7)	67.2 (22.7)	56.7 (25.0)	37.6 (26.5)
Apneas, % OSA events	19.9 (18.1)	22.5 (20.1)	26.8 (20.7)	32.8 (22.7)	43.3 (25.0)	62.4 (26.5)
AHI, events/h	9.5 (2.7)	9.8 (2.8)	21.6 (4.3)	22.0 (4.2)	49.7 (17.4)	55.9 (18.4)
ODI-3%, events/h	8.5 (3.8)	8.8 (3.7)	20.4 (5.8)	20.6 (5.7)	48.3 (18.6)	55.1 (19.1)
Nadir SpO2, %	86 (5)	86 (5)	82 (6)	83 (6)	77 (9)	75 (9)
Time with SpO2 < 90%, min	14 (37)	17 (49)	20 (43)	18 (36)	49 (73)	70 (76)

Data are presented as mean (SD) unless otherwise indicated

Descriptive statistics are presented for the original study sample. Analyses involving the severe OSA subgroup were conducted using inverse probability weighting based on AHI to balance AHI distributions between men and women in the severe OSA groups

AHI = apnea–hypopnea index; PLMI = periodic limb movement index; ODI-3% = oxygen desaturation index based on ≥3% desaturations; N1-N3 = non-rapid eye movement sleep stages 1 to 3; REM = rapid eye movement; TST = total sleep time; SpO2 = peripheral oxygen saturation

**Table 2:** Estimated marginal means for total sleep duration, proportion of time spent in sleep stages, and the total arousal index stratified by sex and OSA severity

Outcome variable	Mild OSA		Moderate OSA		Severe OSA	
	Women (*n* = 274)	Men (*n* = 256)	Women (*n* = 214)	Men (*n* = 266)	Women (*n* = 187)	Men (*n* = 435)
TST, h	6.70 (0.07)	6.43 (0.08)	6.75 (0.09)	6.54 (0.08)	6.66 (0.05)	6.43 (0.05)
N1, % TST	9.94 (0.27)	13.44 (0.37)	11.53 (0.33)	15.57 (0.42)	15.57 (0.30)	21.04 (0.42)
N2, % TST	56.44 (0.72)	55.86 (0.73)	53.75 (0.80)	54.05 (0.71)	52.09 (0.47)	51.66 (0.50)
N3, % TST	15.29 (0.64)	11.89 (0.65)	15.94 (0.71)	11.64 (0.63)	15.07 (0.42)	10.47 (0.44)
REM, % TST	16.93 (0.40)	18.40 (0.40)	18.02 (0.44)	18.07 (0.39)	16.80 (0.26)	16.13 (0.27)
Total arousal index, events/h	8.02 (0.31)	8.45 (0.33)	10.54 (0.45)	11.59 (0.44)	17.29 (0.44)	19.48 (0.52)

Estimated marginal means (SE) were derived from generalized linear models including a sex-by-OSA severity interaction term and adjusted for insomnia, periodic limb movement index, age, and body mass index. Analyses involving the severe OSA subgroup were conducted using inverse probability weighting based on AHI to balance AHI distributions between women and men

N1-N3 = non-rapid eye movement sleep stages 1 to 3; REM = rapid eye movement; TST = total sleep time; OSA = obstructive sleep apnea

### Sleep stage N1 (%TST)

While no significant interaction between sex and OSA severity was observed (Wald χ²(2) = 3.10, *p* = .21; Table 2), the model was re-specified to include main effects only. In the adjusted model, both sex and OSA severity were independently associated with the relative time spent in N1 sleep. Men exhibited a greater proportion of N1 sleep than women (adjusted EMMs [SE]: 16.39 [0.28] vs. 12.13 [0.21] %TST, respectively; Wald χ²(1) = 152.61, *p* < .001). In addition, the proportion of N1 sleep increased progressively with OSA severity (adjusted EMMs [SE]: mild, 11.56 [0.28] %TST; moderate, 13.40 [0.33] %TST; severe, 18.10 [0.29] %TST; Wald χ²(2) = 251.08, *p* < .001).

### Sleep stage N2 (%TST)

The interaction between sex and OSA severity was non-significant (Wald χ²(2) = 0.44, *p* = .80). In the adjusted main effects model, there was no significant sex difference in N2 (adj. EMMs [SE], men vs. women: 53.83 [0.37] vs. 54.14 [0.38] %TST; Wald χ²(1) = 0.35, *p* = .55). OSA severity demonstrated a dose-dependent association with N2 (adj. EMMs [SE], mild vs. moderate vs. severe: 56.15 [0.52] vs. 53.93 [0.53] vs. 51.88 [0.34] %TST; Wald χ²(2) = 45.08, *p* < .001).

### Sleep stage N3 (%TST)

No significant interaction between sex and OSA severity was observed (Wald χ²(2) = 1.26, *p* = .53). The model was therefore re-specified to include main effects only. In the adjusted model, women exhibited a higher proportion of N3 sleep than men (adjusted EMMs [SE]: 15.49 [0.34] vs. 11.27 [0.33] %TST, respectively; Wald χ²(1) = 84.15, *p* < .001). No significant main effect of OSA severity was observed (adjusted EMMs [SE]: mild, 13.58 [0.47] %TST; moderate, 13.79 [0.47] %TST; severe, 12.77 [0.30] %TST; Wald χ²(2) = 3.91, *p* = .14).

### Sleep stage REM (%TST)

A significant interaction between sex and OSA severity was observed (Wald χ²(2) = 10.23, *p* = .006). Pairwise comparisons indicated that men exhibited a higher REM% than women in the mild OSA group (adjusted mean difference: +1.46% points, *p* = .009). No significant sex difference was observed in the moderate OSA group (adjusted mean difference: +0.05% points, *p* = .931). In the severe OSA group, women exhibited a slightly higher REM% than men (adjusted mean difference: +0.66% points), although this difference did not reach statistical significance (*p* = .09). Adjusted EMMs for REM (%TST) by sex and OSA severity are provided in Table 2.

### Total arousal index (events/h)

To further characterize sex differences in sleep fragmentation, we used a generalized linear model with the total arousal index as the outcome. The total arousal index is a measure of sleep disruption and was defined as the number of arousals per hour of sleep, scored according to standard polysomnographic criteria. Higher values indicate more fragmented sleep [17].

No significant interaction between sex and OSA severity was observed (Wald χ²(2) = 1.05, *p* = .59; Table 2). The model was therefore re-specified to include main effects only. In the adjusted model, men exhibited a higher total arousal index than women (adjusted EMMs [SE]: 12.45 [0.25] vs. 11.30 [0.23] events/h, respectively; Wald χ²(1) = 12.12, *p* < .001). Independently, the total arousal index increased progressively with OSA severity (adjusted EMMs [SE]: mild, 8.24 [0.23] events/h; moderate, 11.05 [0.32] events/h; severe, 18.35 [0.34] events/h; Wald χ²(2) = 560.49, *p* < .001).

### Hypopneas (% of apneas and hypopneas)

**Fig. 2:**
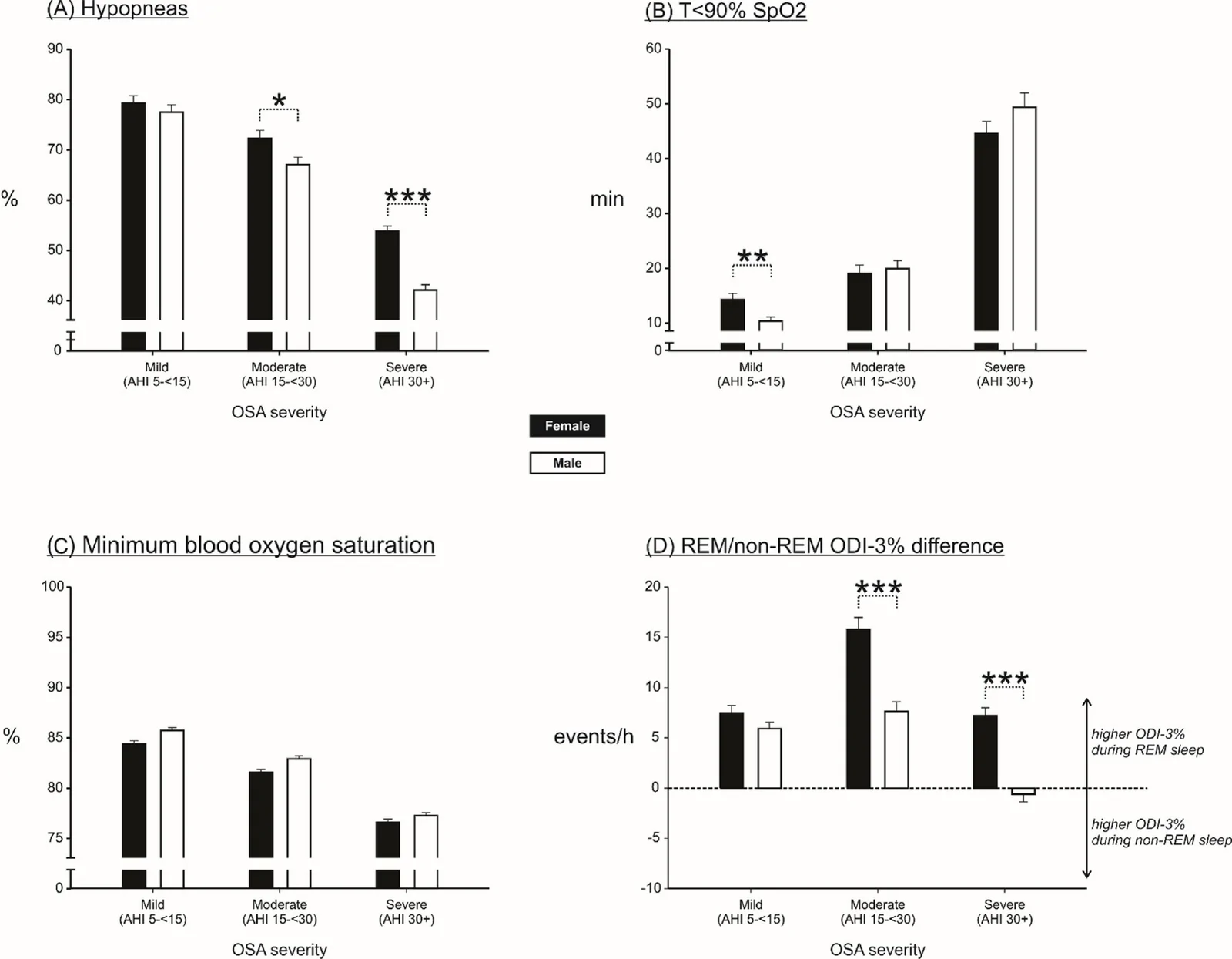
Sex differences in nocturnal oxygenation patterns by OSA severity. Adjusted estimated marginal means for (**A**) hypopnea (% of apneas and hypopneas), (**B**) time with oxygen saturation < 90% during TST (T < 90% SpO2, minutes), (**C**) nadir blood oxygen saturation (%), and (**D**) sleep stage–specific distribution of oxygen desaturation events (REM/non-REM ODI-3% difference). Significant sex-by-severity interactions were observed for hypopnea, T < 90% SpO2, and the sleep stage–specific distribution of oxygen desaturation events, but not for nadir SpO2. Asterisks indicate significant differences between men and women within OSA severity categories and are shown only for outcomes with significant interactions. For main effects of sex and OSA severity on nadir SpO2, see Results section. Models were adjusted for age, BMI, presence of insomnia, periodic limb movement index, TST, and time spent in the supine position. Error bars represent standard errors. *p < .05, **p < .01, ***p < .001

A significant interaction between sex and OSA severity was observed (Wald χ²(2) = 18.38, *p* < .001). As shown in Fig. 2A, no significant sex difference in the relative proportion of hypopneas was observed in mild OSA (adjusted mean difference [SE] for men minus women: −1.81 [2.07] percentage points; *p* = .38). In contrast, men exhibited a lower proportion of hypopneas than women in both moderate OSA (adjusted mean difference [SE]: −5.19 [2.18] percentage points; *p* = .02) and severe OSA (adjusted mean difference [SE]: −11.75 [1.43] percentage points; *p* < .001). Thus, as OSA severity increased, a progressively greater proportion of respiratory events were classified as hypopneas in women relative to men.

### T < 90% SpO2 (min)

The interaction between sex and OSA severity was significant (Wald χ²(2) = 11.39, *p* = .003). As shown in Fig. 2B, in mild OSA, men spent significantly less time below 90% SpO2 than women (adjusted mean difference [SE] for men minus women: −3.94 [1.33] min, *p* = .003). In moderate OSA, no significant sex difference in T < 90% SpO2 was observed (adjusted mean difference [SE]: +0.89 [2.16] min, *p* = .68). Similarly, in severe OSA, men spent more time below 90% SpO2 than women; however, this difference did not reach statistical significance (adjusted mean difference [SE]: +4.78 [3.52] min, *p* = .18).

### Nadir SpO2 (%)

No significant interaction between sex and OSA severity was observed for nadir SpO2 (Wald χ²(2) = 1.59, *p* = .45; Fig. 2C). The model was therefore re-specified to include main effects only. In the adjusted model, women exhibited lower nadir SpO2 values than men, irrespective of OSA severity (adjusted EMMs [SE]: 80.92 [0.22]% vs. 81.91 [0.19]%, respectively; Wald χ²(1) = 10.82, *p* = .001). Thus, women reached lower minimum oxygen saturation levels during sleep than men. As expected, nadir SpO2 decreased progressively with increasing OSA severity (adjusted EMMs [SE]: mild, 85.06 [0.23]%; moderate, 82.26 [0.25]%; severe, 76.93 [0.22]%; Wald χ²(2) = 614.55, *p* < .001).

### REM/non-REM difference in oxygen desaturation events (events/h)

Because oxygen desaturation events are known to occur predominantly during REM sleep in milder OSA, but increasingly extend into non-REM sleep as OSA severity progresses [18], we next examined whether this pattern differed between women and men. The ODI-3%, defined as the number of oxygen desaturations of at least 3% per hour of sleep [16, 17], was calculated separately for REM and non-REM sleep. To quantify the relative predominance of desaturation events across sleep stages, we calculated the difference between ODI-3% during REM sleep and ODI-3% during non-REM sleep. Positive values indicate that 3% desaturation events occurred more frequently during REM sleep, whereas negative values indicate that 3% desaturation events occurred more frequently during non-REM sleep.

A significant interaction between sex and OSA severity was observed (Wald χ²(2) = 14.39, *p* < .001; Fig. 2D). In mild OSA, no significant sex difference was observed in the relative predominance of REM-related 3% desaturation events (adjusted mean difference [SE] for men minus women: −1.54 [1.46], *p* = .292). In contrast, women exhibited a greater REM predominance of 3% desaturation events than men in both moderate OSA (adjusted mean difference [SE]: −8.15 [1.55], *p* < .001) and severe OSA (adjusted mean difference [SE]: −7.77 [1.03], *p* < .001). Within women, the difference between ODI_REM_ and ODI_non−REM_ remained positive across all OSA severity categories, indicating a persistent predominance of 3% desaturation events during REM sleep. In contrast, among men, this difference approached zero in severe OSA, suggesting that 3% desaturation events became more evenly distributed across REM and non-REM sleep.

### Sensitivity analyses

Corresponding analyses performed in the unweighted full sample (*n* = 1,632) are presented in Supplementary Table S2. The overall pattern of findings was like that observed in the primary IPW analyses. While several sex × OSA severity interactions were attenuated after weighting for AHI, the principal findings regarding respiratory event composition and the sleep-stage distribution of oxygen desaturation events remained unchanged.

## Discussion

Understanding how OSA differs between women and men is important for characterizing the heterogeneity of the disorder. Using a large clinical cohort and detailed polysomnographic measures, we identified sex differences in both sleep architecture and nocturnal oxygenation that were not fully captured by conventional OSA severity classification based on AHI.

Women exhibited a more preserved sleep architecture than men across OSA severity categories. Specifically, women spent less time in light non-REM sleep (N1), more time in deep non-REM sleep (N3), and experienced fewer sleep disruptions, as reflected by a lower total arousal index. In contrast, OSA severity itself was associated with progressively lighter and more fragmented sleep, irrespective of sex. These findings are consistent with previous reports suggesting that women may maintain more consolidated sleep despite the presence of OSA [8, 11]. Whether such differences contribute to sex-specific symptom profiles or daytime functioning remains unclear and warrants further investigation.

Sex differences were also observed in the respiratory characteristics of OSA. Women exhibited a greater relative predominance of hypopneas compared with men, particularly in moderate and severe OSA. This finding is consistent with previous observations that respiratory events in women may be characterized by less complete upper-airway obstruction than in men [8–11]. However, the present study was not designed to directly evaluate the physiological mechanisms underlying these differences, and this interpretation should therefore be considered speculative.

With respect to nocturnal oxygenation, women exhibited lower nadir oxygen saturation values than men across OSA severity categories. Women also showed a greater tendency for 3% oxygen desaturation events to occur during REM sleep, particularly in moderate and severe OSA. In contrast, 3% desaturation events among men appeared to become more evenly distributed throughout the night as OSA severity increased. These findings suggest that the sleep-stage distribution of respiratory disturbances may differ between women and men.

Although the present observational study cannot determine the biological mechanisms underlying this sex-specific pattern, several physiological factors may contribute. Women generally have lower lung volumes, including functional residual capacity, than men. Consequently, the pulmonary oxygen reserve available during periods of airflow limitation may be smaller, potentially resulting in more pronounced oxygen desaturations during respiratory events. This effect may be particularly relevant during REM sleep, when muscle tone is markedly reduced and respiratory events may be associated with greater impairment of ventilation [18]. In addition, women exhibited a greater predominance of hypopneas than men, raising the possibility that sex differences in upper-airway obstruction or ventilatory control contribute to the observed REM-related desaturation pattern. However, because measures of lung volume, upper-airway muscle activity, and ventilatory control were not available, these explanations remain speculative and require direct investigation in future studies.

Regardless of the mechanisms underlying these observations, the present findings demonstrate that women and men with similar OSA severity, as defined by AHI, may nevertheless differ in sleep architecture, respiratory event composition, and the sleep-stage distribution of nocturnal oxygen desaturation. Together, these findings highlight sex-specific dimensions of OSA physiology that are not fully captured by AHI alone.

### Limitations

Several limitations should be considered when interpreting these findings. First, because men and women differed in AHI within the severe OSA category, IPW based on AHI was applied to balance OSA severity between sexes in the severe OSA groups. Although this approach substantially reduced the AHI imbalance, residual confounding related to OSA severity cannot be entirely excluded.

Second, the present study examined a broad range of sleep architecture and respiratory outcomes, including multiple sex-by-OSA severity interactions and post hoc comparisons. These analyses were designed to provide a comprehensive characterization of sex differences across several domains of sleep and breathing and should therefore be considered exploratory in nature. Consequently, the possibility of Type I error due to multiple testing cannot be excluded, and the findings should be interpreted with appropriate caution until replicated in independent cohorts.

A further limitation is the lack of information on several potentially relevant confounding factors. Smoking is associated with upper airway inflammation, altered ventilatory control, and increased sleep fragmentation [19, 20], and may therefore contribute to sex differences in sleep and sleep-breathing patterns. In addition, information on menopausal status, hormone replacement therapy, alcohol consumption, and certain comorbid conditions were not available. These factors may influence sleep architecture, respiratory event characteristics, and nocturnal oxygenation and could therefore contribute to some of the observed sex differences. Given that the mean age of women ranged from 58 to 65 years across OSA severity categories, a substantial proportion of the female cohort was likely post-menopausal. Consequently, the observed sex differences may primarily reflect the characteristics of middle-aged and older women with OSA and may not be fully generalizable to pre-menopausal women. In addition, the cohort consisted almost exclusively of Han Chinese individuals. Although OSA in East Asian populations may be influenced to a greater extent by craniofacial anatomical factors than in some Western populations [14, 15], several of the present findings are broadly consistent with observations from Western cohorts [8–11], suggesting that some sex-specific features of OSA may extend across populations. Nevertheless, the extent to which these findings generalize to populations with different ethnic backgrounds, body habitus, and OSA phenotypes remains uncertain. Future studies incorporating more detailed clinical and hormonal information and including diverse populations will be important to further clarify the mechanisms underlying sex differences in OSA presentation and severity and to determine the generalizability of these findings.

### Perspectives and significance

Because this study was observational, the clinical implications of these findings remain uncertain. Nevertheless, the results suggest that physiological characteristics beyond AHI may provide additional information regarding the expression of OSA in women and men. Future studies should determine whether incorporating such measures can improve OSA phenotyping, risk stratification, diagnostic assessment, or prediction of treatment response. Mechanistic studies are also needed to establish the biological factors underlying the observed sex differences.

## Supplementary Information

Below is the link to the electronic supplementary material.

Supplementary Material 1.

## Data Availability

The data used for this study are available upon reasonable request from Meina Wu, Department of Neurology and Sleep Medical Center, Fujian Provincial Governmental Hospital, Fuzhou, China (meinawu@163.com).
