# Supplementary material for: Sex differences in sleep architecture and nocturnal oxygenation across obstructive sleep apnea severity: an observational hospital-based study
Source: Biol Sex Differ. 2026 Jul 7;17:160. doi: 10.1186/s13293-026-00951-4 (PMC13625502; doi:10.1186/s13293-026-00951-4)
Supplement: Supplementary file 1 [file 13293_2026_951_MOESM1_ESM.docx]

| **Supplementary Table S1.** Model specifications and outlier exclusions for generalized linear models (initial sample size = 1,632) | | | | | |
| --- | --- | --- | --- | --- | --- |
| **Dependent variable** | **GLM distribution/link** | **Confounders** | **Outliers removed (\|Std. deviance residual\| > 3), n** | | **Final sample (n)** |
|  |  |  | Men  (mild/moderate/severe OSA) | Women  (mild/moderate/severe OSA) |  |
| **Total sleep duration, sleep stages, and total arousal index** | | |  |  |  |
| TST | Normal distribution, identity link | Insomnia; PLMI; Age; BMI | 0/0/1 | 0/0/0 | 1,631 |
| N1 (% TST) | Gamma distribution, log link |  | 0/0/1 | 1/1/2 | 1,627 |
| N2 (% TST) | Normal distribution, identity link |  | 2/2/2 | 3/3/0 | 1,620 |
| N3 (% TST) | Normal distribution, identity link |  | 2/3/3 | 4/3/1 | 1,616 |
| REM (% TST) | Normal distribution, identity link |  | 2/2/0 | 2/0/1 | 1,625 |
| Total arousal index (events/h) | Gamma distribution, log link |  | 0/0/1 | 2/0/2 | 1,627 |
|  |  |  |  |  |  |
| **Hypoxemic burden** |  |  |  |  |  |
| Hypopneas (% of apneas and hypopneas) | Normal distribution, identity link | Insomnia; PLMI; Age; BMI; TST; Supine sleep time | 2/0/0 | 0/0/0 | 1,630 |
| Time with SpO2 <90% (min) **^A^** | Gamma distribution, log link |  | 8/0/0 | 3/2/0 | 1,619 |
| Nadir SpO2 (%) **^B^** | Normal distribution, identity link |  | 1/2/10 | 2/2/5 | 1,610 |
| ODI-3% REM/non-REM difference | Normal distribution, identity link |  | 0/0/3 | 1/1/5 | 1,622 |
| **^A^** Because the outcome variable contained zero values, 1 was added prior to modeling.  **^B^** The robust estimator was used to reduce the influence of remaining extreme values on standard errors and test statistics.  Outliers were identified using standardized deviance residuals and excluded if residuals fell outside the range of −3 to 3. This procedure was applied consistently across all outcome variables. Exclusions were based on model residuals rather than clinical implausibility of the observed values.  Abbreviations: N1-N3 = non-rapid eye movement sleep stages 1 to 3; REM = rapid eye movement; PLMI = periodic limb movement index; ODI-3% = oxygen desaturation index based on ≥3% desaturations; TST = total sleep time; SpO2 = peripheral oxygen saturation; BMI = body mass index. | | | | | |
|  | | | | | |

| **Supplementary Table S2.** P-values from adjusted generalized linear models examining main effects of sex and OSA severity and their interaction in the unweighted full sample (n = 1,632) | | | | | | | | |
| --- | --- | --- | --- | --- | --- | --- | --- | --- |
| **Dependent variable** | **GLM distribution/link** | **Confounders** | **Main effect model, p-value** | | | | **Interaction, p-value** | |
|  |  |  | **Sex** | | **OSA severity** | | **Sex-by-OSA severity** | |
| **Total sleep duration, sleep stages, and total arousal index** | | | |  | |  | |  |
| TST | Normal distribution, identity link | Insomnia; PLMI; Age; BMI | < .001 | | .37 | | .88 | |
| N1 (% TST) | Gamma distribution, log link |  | < .001 | | < .001 | | < .001 | |
| N2 (% TST) | Normal distribution, identity link |  | .33 | | < .001 | | .26 | |
| N3 (% TST) | Normal distribution, identity link |  | < .001 | | < .01 | | .36 | |
| REM (% TST) | Normal distribution, identity link |  | .61 | | < .001 | | < .01 | |
| Total arousal index (events/h) | Gamma distribution, log link |  | < .001 | | < .001 | | .001 | |
|  |  |  |  | |  | |  | |
| **Hypoxemic burden** |  |  |  | |  | |  | |
| Hypopneas (% of apneas and hypopneas) | Normal distribution, identity link | Insomnia; PLMI; Age; BMI; TST; Supine sleep time | < .001 | | < .001 | | < .001 | |
| Time with SpO2 <90% (min) **^A^** | Gamma distribution, log link |  | .01 | | < .001 | | .02 | |
| Nadir SpO2 (%) **^B^** | Normal distribution, identity link |  | .14 | | < .001 | | .03 | |
| ODI-3% REM/non-REM difference | Normal distribution, identity link |  | < .001 | | < .001 | | < .001 | |
| **^A^** Because the outcome variable contained zero values, 1 was added prior to modeling.  **^B^** The robust estimator was used to reduce the influence of remaining extreme values on standard errors and test statistics.  Abbreviations: N1-N3 = non-rapid eye movement sleep stages 1 to 3; REM = rapid eye movement; PLMI = periodic limb movement index; ODI-3% = oxygen desaturation index based on ≥3% desaturations; TST = total sleep time; OSA = obstructive sleep apnea; SpO2 = peripheral oxygen saturation; BMI = body mass index. | | | | | | | | |
